# A Lycopodium Cure

**Published:** 1891-06

**Authors:** H. Goullon

**Affiliations:** Weimar


### A LYCOPODIUM CURE.

Dr. H. Goullon, Weimar.

A. H. Z., 13, '91.

H., fifty-five years old, took sick last February with a severe neuralgia, which he had repeatedly experienced during last spring. It begins as a dull pressure in the right side below the last rib near the vertebral column, the increasing pains radiate forward into the abdomen, simulating enteralgia, or into the back. Characteristic is the increasing impossibility to lie down, he turns and twists and finds the most relief in the knee-elbow position. Sleep is impossible, as the pains continue nearly all through the night. Micturition and vomiting of acid and bitter stuff, taste bilious and bitter, with disgust for all food.

As mental complication may be mentioned excessive irritability, as our otherwise gentlemanly patient swears like a trooper, a thing unusual with him. Constipation for several days, till intestinal functions show returning activity again by the passage of some inodorous flatus. He probably caught cold during the inclement snow weather, aggravated by an acute gastric catarrh, so that digestion is at a low ebb, and he is disgusted with himself and wishes to be left alone, moaning continually and damning his pains and every other thing. On the second day of his suffering a complete acute vesical catarrh set in, with fever and nocturnal palpitations (probably from the use of cold beer), or by radiation from the original point of the disease. The patient had to get up thirty or forty times during the night, with tenesmus and intense burning pain during and after micturition, as if hot lead passed through the urethra. The scanty urine was murky, brown, dirty-red, thick, and of a moldy odor. Lycopodium$^{12c}$, six drops in half a glass of water, was prescribed, a teaspoonful every three hours, producing very soon a copious, though still painful urination, which ceased with the copious passage of more urine, and soon old Richard was himself again.

Compare Allen's *Encyclopœdia*, Lycopodium, Symptoms 8, 9, 30, 62, 70, 89, 90, in relation to mind; 1030, 1070, 1112, 1133, 1215–1220, stomach and liver; 1400, colic, especially in transverse colon; 1570, 1583–1590, 1622, etc., in regard to micturition. As usual, the mental symptoms aided to elucidate the bodily symptoms, and in their totality the simillimum was easily found by such a good prescriber as Goullon is known to be.

